# Pineapple core from the canning industrial waste for bacterial cellulose production by *Komagataeibacter xylinus*

**DOI:** 10.1016/j.heliyon.2023.e22010

**Published:** 2023-11-03

**Authors:** Efri Mardawati, Devi Maulida Rahmah, Nova Rachmadona, Elen Saharina, Tanti Yulianti Raga Pertiwi, Siti Aisyah Zahrad, Wahyu Ramdhani, Yoice Srikandace, Diah Ratnaningrum, Een Sri Endah, Dian Andriani, Kuan Shiong Khoo, Khatarina Meldawati Pasaribu, Rahmat Satoto, Myrtha Karina

**Affiliations:** a Department of Agro-Industrial Technology, Universitas Padjadjaran, Jatinangor, 45365, Indonesia; b Research Collaboration Center for Biomass and Biorefinery between BRIN and Universitas Padjadjaran, Jatinangor, West Java, 45363, Indonesia; c Department of Chemistry, Faculty of Mathematics and Natural Sciences, Universitas Padjadjaran, Jatinangor, West Java, 45363, Indonesia; d Faculty of Mathematics and Natural Sciences, Bandung Institute of Technology, Jl. Ganesha No.10, Bandung, 40132, Indonesia; e School of Life Sciences and Technology ITB, Bandung Institute of Technology, Jl. Ganesha No.10, Bandung, 40132, Indonesia; f Research Center for Environmental and Clean Technology, National Research and Innovation Agency, Kompleks BRIN, Jalan Sangkuriang-Cisitu, Bandung, 40135, Indonesia; g Research Center for Applied Microbiology, National Research and Innovation Agency, Cibinong Science Center, Jl. Raya Bogor Km. 46, Bogor, Indonesia; h Department of Chemical Engineering and Materials Science, Yuan Ze University, Taoyuan, Taiwan; i Research Center for Biomass and Bio-product, National Research and Innovation Agency, Jalan Raya Jakarta-Bogor, Km. 46, Cibinong, 16911, Indonesia; j Research Collaboration Center for Nanocellulose, BRIN - Andalas University, Padang, 25163, Indonesia

**Keywords:** Bacterial nano cellulose, Pineapple core, Pineapple canning industrial waste, Characterization, Carbon source, Low cost

## Abstract

To address the high production cost associated with bacterial cellulose (BC) production using the Hestrin-Schramm (HS) medium, alternative agricultural wastes have been investigated as potential low-cost resources. This study aims to utilize pineapple core from pineapple canning industry waste as a carbon source to enhance the bacterial growth of *Komagataeibacter xylinus* and to characterize the physical and mechanical properties of the resulting BC. To assess growth performance, commercial sugar at concentrations of 0, 2.5, and 5.0 % (w/v) was incorporated into the medium. Fermentation was conducted under static conditions at room temperature for 5, 10, and 15 days. The structural and physical properties of BC were characterized using SEM, FTIR, XRD, and DSC. With the exception of crystallinity, BC produced from the pineapple core medium exhibited comparable characteristics to BC produced in the HS medium. These findings highlight the potential of utilizing pineapple core, a byproduct of the canning industry, as an economically viable nutrient source for BC production.

## 1 Introduction

Bacterial cellulose (BC) is a cellulose produced during acetic fermentation in a medium of Acetobacter xylinus, now commonly known as Komagataeibacter *xylinus* [1]. As a natural polymer, the physicochemical properties of BC are superior to those found in plant cellulose but have a similarly molecular formula [2]. Purification of BC is not necessary such as plant cellulose because it is free of lignin and hemicellulose. Additionally, since BC occurs in nano-sized cellulose fiber dimension, high crystallinity [3], high Young's modulus [4], and biocompatibility [5], BC is fascinating and regarded as an excellent renewable biomaterial [6]. Various effort has been reported to use BC for sustainable and functional material such as electromagnetic interference shielding [7], osmotic energy harvesting [8], colorimetric sensor [9]removal of bisphenol A [10], dental application [11], medical application [12], dairy industry [13], wastewater treatment [14], textile material [15] anti-microbial food packaging [16].

Although it possesses remarkable characteristics and is prospective for a broader range of commercial applications, the BC is somewhat costly to fabricate due to the use of pricy Hestrin-Schramm (HS) culture media and the low yield of the use of bacteria strain [17]. It was calculated that the BC costs around 30 % of the total production expense [18]. Hence, numerous agricultural industry by-products decreased the BC production cost and developed the yield. In terms of agro-industry waste utilization, several evaluations have been studied, such as the use of corn stover [19], waste of date syrup and food-grade sucrose industry [6], rotten guava [20], oat hulls [21], confectionary wastes [22], even from kitchen waste [23].

Another potential agro-industrial waste includes pineapple residues obtained from the pineapple canning industrial waste, which generates enormous amounts of solid waste [24]. This waste contains protein, fiber, vitamins, minerals, amino acids, phenolic compounds, oligosaccharides, and polysaccharides [25]. Because carbohydrates are found in pineapple waste, thus it can be used as a carbon source for microbial growth during fermentation [19]. Additionally, it has the potential as a source of growth media for *K. xylinus*.

**Fig. 1:**
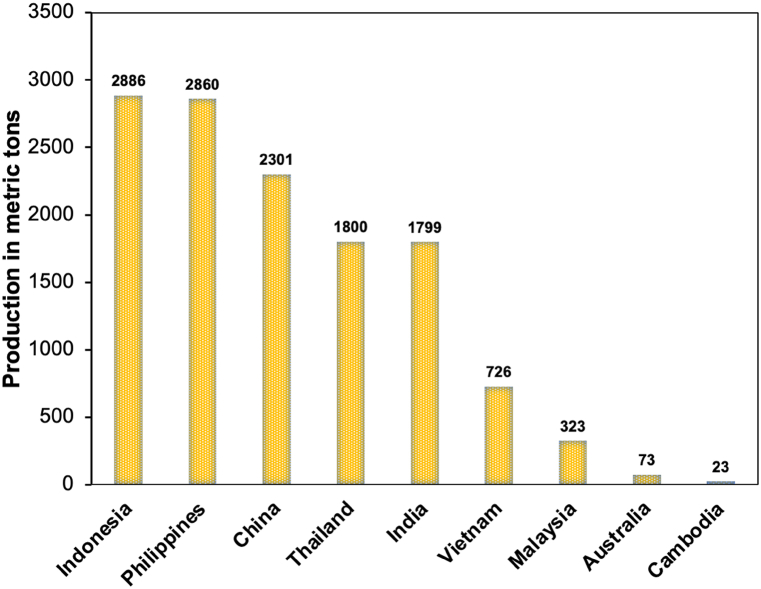
Pineapple producing country.

Moreover, using these wastes would be a great opportunity to create new products while reducing the amount of waste generated by pineapple canning industrial waste [26]. The pineapple canning industrial waste was also used for the production of biogas [27], ethanol [28], lactic acid [29], vanillin and vanillic acid [30], and polyhydroxybutyrate. The use of pineapple industrial wastes such as peel, crown, and its mixture with the core has been studied for BC production [[31], [32], [33]]. However, the utilization of core from the pineapple canning industrial waste for BC production has not been reported. Meanwhile, Indonesia is the largest pineapple producer in the Asia Pacific with total production of 2.886.416 tones [34] (Fig. 1). From the pineapple processing, it is calculated that the core composes ca. 7 % of the total waste [35]. Additionally, the pineapple core contains 41.1–43.5 % of cellulose, 28.5 % of hemicellulose, 5.78 % of lignin, and 0.85 % of protein [36]. It was also reported that the core containing 84.90 % of moisture content, 83.03 % of carbohydrate, crude fat of 2.35–4.78 %, and the crude fiber content of 42.02 % [37]. The core also rich of glucose and fructose and could be invaluable in fermentation process [38]. Therefore, the core of pineapple core may be used as a raw material for BC production.

This research focuses on the potential of pineapple core from the canning industrial waste, which could provide economical source of nutrients to boost BC production from the available resources. The microstructure of BC synthesized by *K. xylinus* is described and compared to BC synthesized in a standard HS medium.

## 2 Materials and methods

### 2.1 Materials

Pineapple core was obtained from the by-product of the pineapple canning industry, small and medium enterprises, Alamsari Serba Nanas in West Java, Indonesia. The pineapple (*Ananas comosus*) was obtained from the Subang Plantation (S 6° 34′ 16.6224″, E 107° 45′ 34.8876″) and harvested 6 months after its cultivation, in March 2022. The main component of the pineapple core was analyzed for their ash content (SNI 01.28911), total carbohydrate (AOAC 982.14), total fat (AOAC 925.12), crude protein (AOAC 992.15), and water content (SNI 01.28911) as shown in Table 1. The starter bacteria as a working inoculum were *K. xylinus* strain CGMCC 2955 purchased from a nata de coco small industry in Cianjur, West Java, Indonesia [39].

**Table 1:** Main component of pineapple core.

Component	%
Ash	0.6
Carbohydrate	4.3
Fat	0.4
Protein	0.1
Water content	94.6

A typical synthetic Hestrin-Schramm (HS) medium that is frequently used for BC production in laboratories was also employed for comparison. The modified HS medium was prepared containing glucose 25 g/L, peptone 5 g/L, yeast extract 5 g/L, citric acid 1.15 g/L, and disodium hydrogen phosphate 2.7 g/L [40]. Local commercial table sugar (Gulaku) was used to replace the glucose commonly used in the HS medium and purchased from the local supermarket, and used without further treatment or purification.

### 2.2 BC production and harvesting

The pineapple core was put in a beaker glass and added with water. The ratio of fresh pineapple core to distilled water of 1:2 (w/v). To compare with glucose used in HS medium, various sugar concentrations of 0, 2.5, and 5.0 % **(w/v),** respectively were added into the mixture and boiled for 30 min. After cooling and filtering, the filtrate was added with glacial acetic acid until its pH to 4. The fermentation was prepared in 500 mL beaker glass containing 200 mL of medium which is then inoculated with 40 mL of commercial culture of bacterial starter and incubated for 5, 10, and 15 days under static condition at room temperature.

At the end of the fermentation phase, the BC pellicle was taken from the medium and washed with water to remove the leftover medium. As described previously, to get rid of impurities or the leftover of bacteria, the pellicle was boiled in water for 10 min then cast off. This process was repeated until its pH was the same as the pH of water [39]. To remove the excess of water, the wet BC pellicle was squeezed by filter-vacuum under nylon fabric, followed by microwave oven-drying (300 W, 3 min) and then heat-pressed (125 °C, 30 kgf/cm^2^, 10 min), respectively. The dry weight of BC per liter of medium (g/L) was used to calculate BC yield.

### 2.3 Reducing sugar analysis

Reducing sugar analysis of medium for *K. xylinus* growth was carried out by the Nelson-Somogyi method as described elsewhere [41].

### 2.4 Morphological analysis

The morphology of BC was observed by a Scanning Electron Microscope (SEM) (JEOL JSM IT-300, Japan). The specimens were coated with a thin layer of gold (±10 nm). The SEM micrograph was taken at an acceleration voltage of 20 kV.

### 2.5 Fourier transform infrared (FTIR) spectroscopy

Fourier transform infrared (FT-IR) spectroscopy (Thermo Scientific™ Nicolet™ iS™5 FTIR Spectrometer) with attenuated total reflectance (ATR) mode at wavenumbers ranging from 4000 to 400 cm^**−1**^ was used to characterize the functional group of BC [42].

### 2.6 X-ray diffraction (XRD)

XRD measurements on a Bruker D8 Advance X-ray Diffractometer operating at 40 kV and 30 mA with CuKα radiation as the X-ray source and LYNXEYE XE-T as the detector was performed to determine the crystalline segment of samples. At a scanning rate of 3°/min, samples were scanned in 2θ ranges from 5 to 40°.

### 2.7 Differential scanning calorimetry (DSC)

The DSC value of BC was analyzed by a differential scanning calorimeter (DSC-214 Polyma, Netzsch, Germany). An amount of BC (5–7 mg) was placed in an alumina pan and then heated from 30 to 250 °C with a rate of 5 °C/min, under a nitrogen atmosphere (50 mL/min).

### 2.8 Statistical analysis

Statistical analysis was performed using one-way analysis of variance (ANOVA), followed by the post hoc Tukey's HSD procedure. The differences within group were considered significant at a probability level of 0.05 (p ≤ 0.05). The assumption of equal variances was verified by Brown-Forsythe Test and Welch Test, respectively.

## 3 Results and discussion

The industrial production of BC is inhibited by the growth media cost of roughly 30 % of the total BC production budget when standard nutrients are used [43]. To minimize the raw materials cost, free pineapple core from the pineapple canning industrial waste were replaced for the commercial and pricy HS medium. Utilizing this byproduct could result in more cost-effective and sustainable production in BC. Additionally, the pineapple core was not sterilized in an autoclave resembling the simplicity of the method. As for the after-treatment efficiency, BC was boiled in water compared with soaked in hot 2 % NaOH, as reported previously [44]. The yield of BC produced from various sugar concentrations, fermentation times and its labelling was shown in Table 2.

**Table 2:** Yield of BC from various sugar concentration.

Day-	Sugar concentration (%)	Sample	Mean	SD
5	0	BC 0/5	0.56^a^	0,04
	2.5	BC 2.5/5	1.00^b^	0,04
	5.0	BC 5/5	1.65^c^	0,04
	2.5	HS 2.5/5	0.51^a^	0,01
10	0	BC 0/10	0.9^a^	0,02
	2.5	BC 2.5/10	2.00^b^	0,05
	5.0	BC 5/10	2.00^b^	0,05
	2.5	HS 2.5/10	2.21^c^	0,06
15	0	BC 0/15	2.45^a^	0,06
	2.5	BC 2.5/15	2.49^a^	0,06
	5.0	BC 5/15	2.52^a^	0,05
	2.5	HS 2.5/15	2.55^a^	0,05

Each values represent mean of replicate, alphabets as superscript across.

Rows indicates a significant difference p ≤ 0.05 by Tukey test.

Notes.

BC produced in pineapple core.

HS produced in Hestrin-Schramm medium.

### 3.1 BC yield

Yield is an important aspect in BC production, especially for commercial purposes. Therefore, the utilization of cost-effective media, such as pineapple core, plays a significant role in BC production. The influence of pineapple core supplemented with sugar on BC yield is presented in Table 2. The pineapple core medium used in this experiment is suitable for BC production as it is described here. Our current findings demonstrate that the yield obtained from the medium without sugar addition is comparable to that of the standard high-sugar (HS) medium. This observation can be attributed to the presence of carbohydrates in the pineapple core, which serve as a carbon source for bacterial growth. The pineapple core contains approximately 4.5 % fiber, which further contributes to the availability of carbon sources necessary for bacterial growth, as fibers also serve as carbon sources [[[45], [46]]

**Fig. 2:**
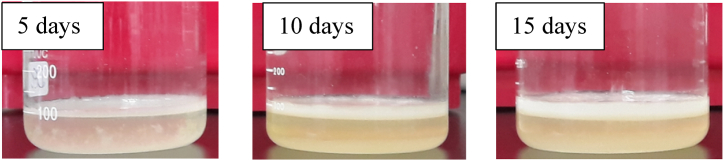
BC production based on the fermentation days.

This achievement highlights the potential of utilizing pineapple core, a by-product of the pineapple canning industry, as an alternative resource for the production using HS medium, which is typically considered costly for BC production. Previous studies have demonstrated comparable yields using pineapple peel extract waste (40 % v/v) as the medium, resulting in a yield of 2.42 g/L when incubated for 6 days at pH 4.5 using *K. xylinus* [47]. Additionally, a slightly higher yield of 3.24 g/L was achieved from pineapple peel by utilizing a 1 % v/v inoculum of *Gluconacetobacter medellinensis* and incubating for 13 days [48]. The gradual increase in BC yield with prolonged fermentation time is consistent with previous findings using *K. xylinus* and various bio-wastes as substrates for BC production [49] (Fig. 2). Statistical analysis indicated significant differences (p < 0.05) in the yields of the pellicles as shown in Table 2.

### 3.2 Analysis of glucose content in pineapple core media

**Fig. 3:**
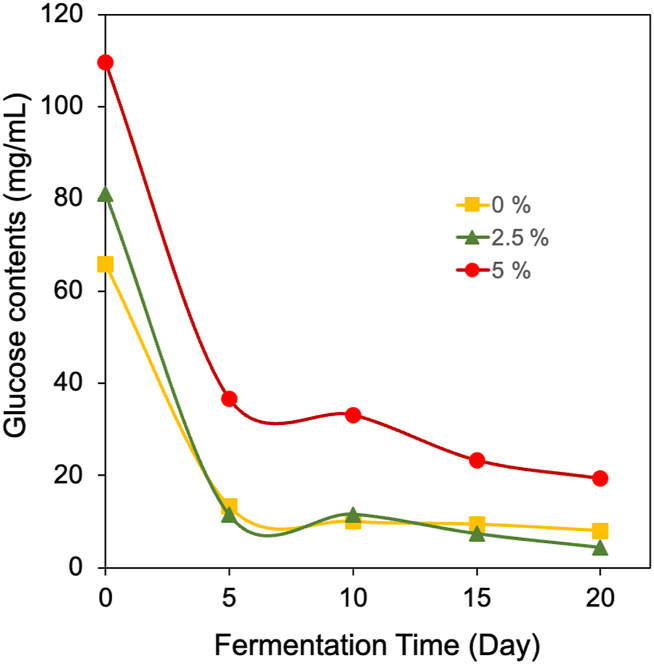
The changes of glucose contents in the medium with no addition of glucose, with 2.5, and 5 % addition of sugar during the fermentation process.

The glucose content of all treatments shown in Fig. 3 decreased significantly during the 5 days of fermentation, indicating that the microorganisms were growing rapidly and therefore a large amount of glucose was consumed. By day- 10, the glucose content increased slightly, possibly due to glucose regeneration from the breakdown of other carbohydrate sources in the waste. After the 10th to the 20th day of fermentation, all graphs showed a tendency for glucose levels to decrease at a slower rate as the bacteria were no longer focused on growing or multiplying. However, this increased BC production started to be observed from day 5 of fermentation as presented in Table 2.

Media without glucose and with 2.5 % glucose addition showed a similar graph of glucose content, however it shows that the sugar consumption rate in medium with 2.5 % sugar content was higher than in medium without sugar addition. The 5-day sugar consumption rate of the fermentation on medium fields 0, 2.5, and 5 % were 10.51, 34.84, and 14.59 mg/mL per day, respectively. Additionally, the oxygen required for *K. xylinus* to carry out additional metabolic or for its growth may be reduced by the formation of a BC layer on the media's surface.

A number of bacterial strains use mannitol, glucose, fructose, sucrose, and other carbon sources as building blocks to generate the biopolymer known as BC as a main metabolite [50]. When bacteria first begin to proliferate, primary metabolites are created as cell mass or number increases [51]. The presence of oxygen in aerobic environments contributes to the metabolic processes going on in addition to serving as a source of sustenance. The oxygen transfer rate, which declines as broth viscosity increases and CO_2_ pressure is followed by BC buildup, determines the rate of BC synthesis [52].

### 3.3 Morphological analysis

**Fig. 4:**
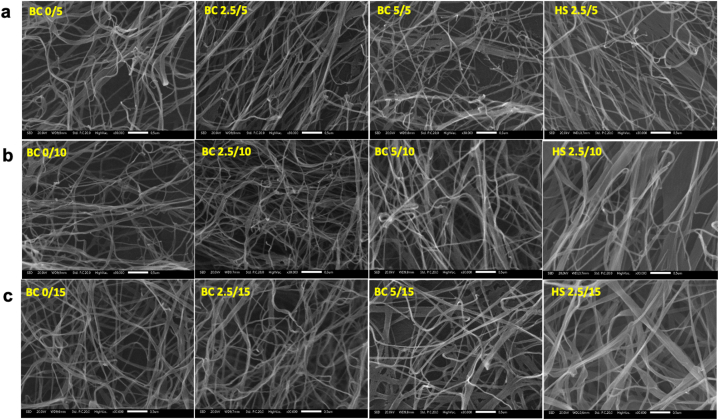
SEM images of BC from pineapple core and HS medium at a) day-5, b) day- 10, and c) day- 15 of fermentation.

The properties of BC are closely related to its morphological characteristics, which play an important role in its assessment. In Fig. 4(a–c), the surface micrograph of BC derived from pineapple core at different sugar concentrations on day 5, 10, and 15 of fermentation is presented. From the observations in Fig. 4(a–c), it is evident that there are no structural differences among the fibers in BC gel formed with or without the addition of sugar. The BC fibers exhibit an irregular three-dimensional network composed of densely packed and disordered fibrils. Interestingly, the BC produced from pineapple core exhibits a heterogeneous three-dimensional network structure similar to that of BC produced in high-sugar (HS) medium. This indicates that the utilization of pineapple core, derived from canning industrial wastes, has the potential to serve as a low-cost growth medium for *K. xylinus* in cellulose production. This finding aligns with a previous report in which BC synthesis was conducted using corn steep liquor [53]. Additionally, although the diameter size may differ, the irregular three-dimensional network of disordered fibrils in BC bears resemblance to bamboo cellulosic fiber [54].

**Fig. 5:**
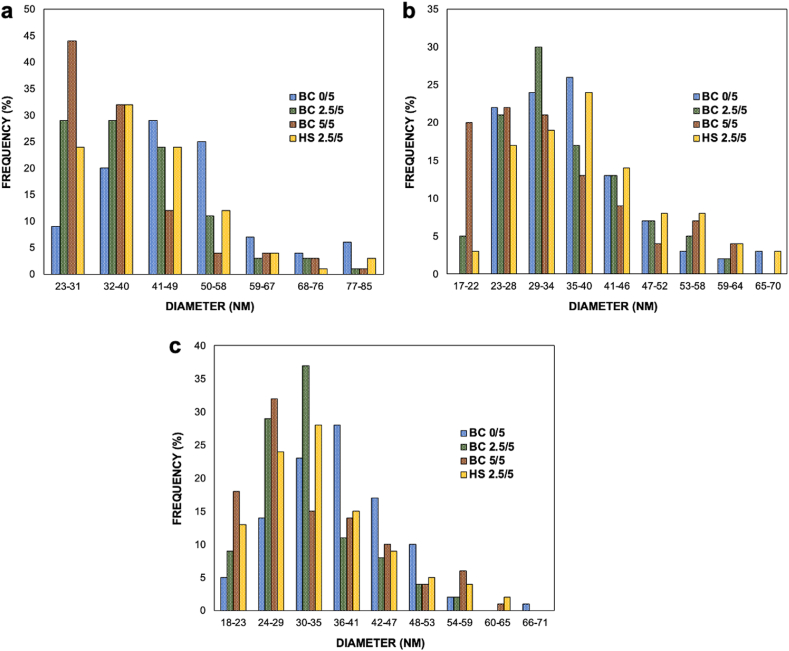
Frequency histogram of BC from pineapple core and HS medium at a) day-5, b) day- 10, and c) day- 15 of fermentation.

The dimension of the nanofibril diameter was observed between BC derived from pineapple waste and nanofibrils obtained from HS medium. The frequency histogram in Fig. 5(a–c) presents the distribution of BC diameter from pineapple waste medium at various sugar concentrations on day 5, 10, and 15 of fermentation. The observed fibril diameters ranged from 23 to 85 nm. The diameter of BC fibers produced in this study is comparable to that of BC obtained from crude distillation waste after oven-drying at 50 °C for one and a half hours [55]. This result suggests that the utilization of pineapple waste as a growth medium for BC production can yield microfibrils with similar diameter characteristics to those obtained from conventional HS medium. The consistent fibril dimensions indicate the potential of pineapple waste as a suitable and cost-effective alternative substrate for BC synthesis.

### 3.4 Fourier transform infrared (FTIR) spectroscopy

**Fig. 6:**
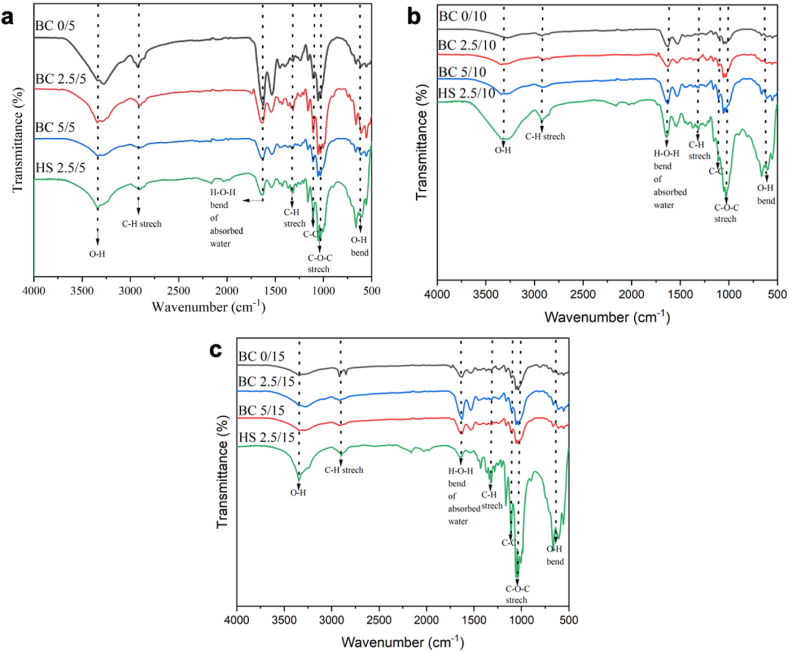
FTIR spectra of BC from pineapple core and HS medium at a) day-5, b) day- 10, and c) day- 15 of fermentation.

Fourier-transform infrared spectroscopy (FTIR) is a suitable technique for analyzing the chemical bonds and specific functional groups in a molecule, providing insights into the alterations induced by fermentation treatment. Fig. 6(a–c) illustrates the FTIR spectra of BC obtained from pineapple core medium at different sugar concentrations on day 5, 10, and 15 of fermentation. The FTIR spectrum exhibits distinct absorption peaks, with the characteristic absorption at 3341 cm^−1^ attributed to the intra and inter O–H stretching vibrations in cellulose I [56] A weaker peak observed around 2918 cm^−1^ corresponds to the C–H stretching of CH_2_ and CH_3_ groups. Furthermore, a strong peak at 1636 cm^−1^ indicates the H–*O*–H bending vibration of water absorption [57]. The absorption at approximately 1107 cm^−1^ is associated with the C–C vibration of the monomer units of polysaccharides [58]. The bands observed in the region of 1030–1060 cm^−1^ are linked to the stretching vibrations of C–*O*–C and C–O bonds [59]. Additionally, the peak at 663 cm^−1^ corresponds to the O–H out-of-phase bending vibration [57]. From the FTIR spectra presented in Fig. 4, it is evident that BC derived from pineapple core exhibits similar absorption features to those obtained from HS medium. This similarity in FTIR spectra indicates a comparable chemical composition and functional groups between BC produced from pineapple core and BC produced from HS medium. Consequently, pineapple core can serve as a low-cost and promising alternative growth medium for BC production, potentially replacing the traditional HS medium.

### 3.5 X-ray diffraction analysis

**Fig. 7:**
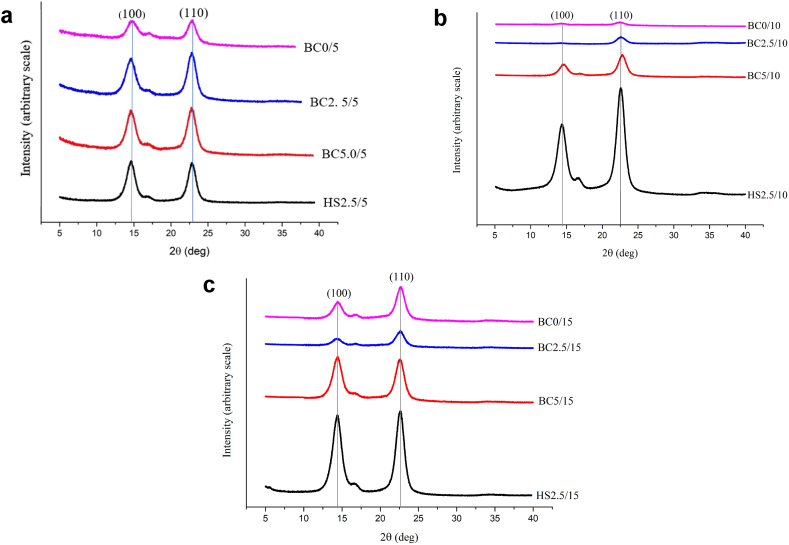
X-ray diffractogram of BC from pineapple core and HS medium at a) day-5, b) day- 10, and c) day- 15 of fermentation.

X-ray diffraction (XRD) analysis is a widely used technique for characterizing the structure and assessing its changes in BC. In Fig. 7(a–c), the XRD curves for BC production in pineapple core medium with varying sugar additions are presented. The XRD pattern of all pineapple core samples exhibits similar patterns, characterized by two prominent peaks at approximately 2θ values of 14.4° and 22.6°, corresponding to the Miller indices of (100) and (110), respectively, which are indicative of cellulose I_α_ structure [60]. The diffractogram obtained in this study closely resembles the XRD pattern of BC produced using *K. xylinus* in kombucha medium [61] and resembles the XRD pattern of cellulose derived from plants [62]. Additionally, Fig. 7(a–c) demonstrates that the diffractogram of BC obtained from pineapple core is nearly identical to that of BC prepared using the HS medium.

These results indicate that the sugar concentration significantly influences the X-ray diffraction of BC. Furthermore, it is noteworthy that the crystal plane of BC membranes obtained from HS medium is similar compared to that of the pineapple core medium. This difference in peak height can be attributed to the variations in nutrient composition present in the HS medium, which might impact the cellulose structure [63]. The XRD analysis confirms that BC produced from pineapple core exhibits a similar structure resembling to that of BC produced using traditional HS medium. The plane structure of BC is influenced by the sugar concentration, with higher diffractogram observed in BC membranes derived from HS medium. These findings shed light on the impact of growth medium composition on the properties of BC, providing valuable insights for further optimization of BC production processes.

### 3.6 Differential scanning calorimetry (DSC)

**Fig. 8:**
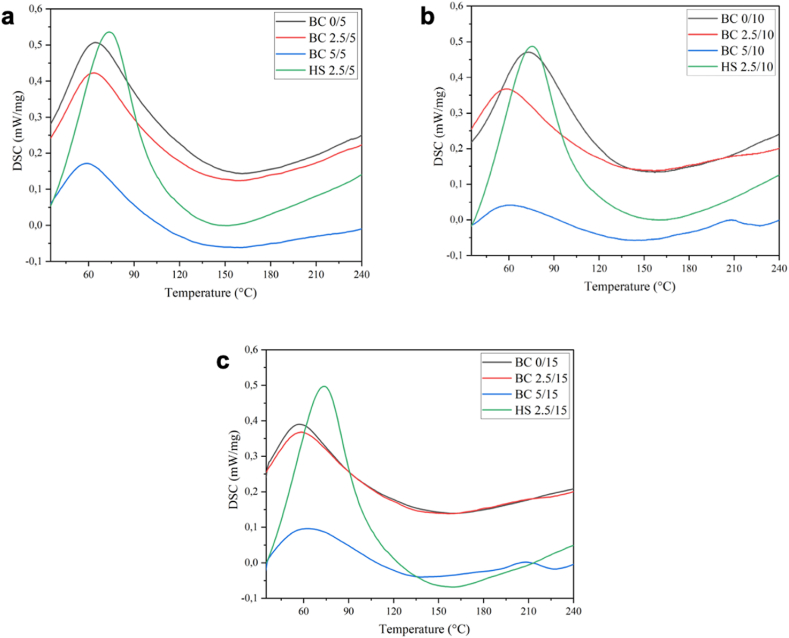
DSC of BC from pineapple core and HS medium at a) day-5, b) day- 10, and c) day- 15 of fermentation.

Differential scanning calorimetry (DSC) is a thermodynamic technique used to directly assess the heat energy absorbed or released by a sample as it undergoes temperature changes. Fig. 8(a–c) presents the DSC analysis of BC production in pineapple core medium with varying sugar concentrations. As depicted in Fig. 8(a–c), all BC samples exhibit similar patterns of broad endothermic and exothermic peaks. The observed endothermic peaks in the temperature range of 30–105 °C for all samples can be attributed to water dehydration [64]. This behavior is commonly observed in cellulosic materials due to the interaction between water molecules and non-substituted hydroxyl groups. The broad endothermic peak at approximately 65 °C in BC derived from pineapple core may be attributed to random chain breakage [65]. On the other hand, the slightly sharper and higher endothermic peak at around 74 °C is characteristic of BC produced from HS medium. The higher peak temperature suggests a more homogeneous distribution of heat and stronger chain-to-heat interactions. Additionally, a distinct and significant exothermic peak observed at approximately 155 °C between the two DTA endothermic peaks could be attributed to chain breaking termination [65]. This DSC analysis reveals similar thermal behavior in BC samples obtained from pineapple core and HS medium. The observed endothermic peaks associated with water dehydration and chain breakage indicate typical characteristics of cellulosic materials. The sharper and higher endothermic peak observed in HS-based BC suggests a more robust chain-to-heat interaction. The presence of a prominent exothermic peak between the endothermic events further indicates the occurrence of chain breaking termination. These findings provide valuable insights into the thermal properties and behavior of BC produced from pineapple core, demonstrating its potential as a viable alternative to HS medium in BC production.

In current study, the BC production using pineapple core medium was successfully achieved. The cost of the pineapple from the canning industrial wastes was calculated, revealing lower production cost of 68 % comparing with HS medium. The results shows the potential of pineapple core utilization for BC production. Carbohydrate in pineapple core was used as a carbon source to replace the glucose sources in HS medium.

## 4 Conclusion

In conclusion, the findings of this study highlight the potential of utilizing low-cost substrates, specifically pineapple core derived from canning industrial waste, as an alternative medium for BC production under static conditions. These underutilized by-products serve as a viable replacement for HS medium. The addition of sugar to the pineapple core media effectively stimulated BC pellicle formation, with fermentation time playing a crucial role in this process. Importantly, the structural, physical, and thermal characteristics of BC produced from the pineapple core medium were found to be comparable to those obtained from the HS medium. This demonstrates that pineapple core can serve as a sustainable and cost-effective alternative substrate for BC production, offering comparable BC quality to the conventional HS medium. These findings contribute to the advancement of BC production processes by utilizing agricultural waste and promoting a more sustainable approach in the field of biomaterials.

## Data availability

Data will be made available on request.

## CRediT authorship contribution statement

**Efri Mardawati:** Formal analysis, Methodology, Project administration, Supervision, Validation. **Devi Maulida Rahmah:** Project administration, Writing – review & editing. **Nova Rachmadona:** Formal analysis, Visualization, Writing – original draft, Writing – review & editing. **Elen Saharina:** Investigation, Methodology. **Tanti Yulianti Raga Pertiwi:** Investigation. **Siti Aisyah Zahrad:** Investigation. **Wahyu Ramdhani:** Investigation. **Yoice Srikandace:** Methodology, Validation. **Diah Ratnaningrum:** Investigation. **Een Sri Endah:** Data curation, Supervision. **Dian Andriani:** Formal analysis, Resources. **Kuan Shiong Khoo:** Formal analysis, Supervision. **Khatarina Meldawati Pasaribu:** Formal analysis, Visualization. **Rahmat Satoto:** Formal analysis, Methodology, Validation. **Myrtha Karina:** Conceptualization, Formal analysis, Funding acquisition, Methodology, Project administration, Resources, Supervision, Validation, Writing – original draft, Writing – review & editing.

## Declaration of competing interest

The authors declare that they have no known competing financial interests or personal relationships that could have appeared to influence the work reported in this paper.

## References

[1] Du R., Zhao F., Peng Q., Zhou Z., Han Y. (2018). Production and characterization of bacterial cellulose produced by *Gluconacetobacter xylinus* isolated from Chinese persimmon vinegar. Carbohydr. Polym..

[2] Ul-Islam M., Khan T., Park J.K. (2012). Water holding and release properties of bacterial cellulose obtained by in situ and ex situ modification. Carbohydr. Polym..

[3] Jung H. (2010). Influence of glycerol on production and structural – physical properties of cellulose from *Acetobacter sp*. V6 cultured in shake flasks. Bioresour. Technol..

[4] Nishi Y. (1990). The structure and mechanical properties of sheets prepared from bacterial cellulose Improvement of the mechanical properties of sheets and. J. Mater. Sci..

[5] Islam M.U., Ullah M.W., Khan S., Shah N., Park J.K. (2017). Strategies for cost-effective and enhanced production of bacterial cellulose. Int. J. Biol. Macromol..

[6] Faria M., Mohammadkazemi F., Aguiar R., Cordeiro N. (2022). Agro-industrial byproducts as modification enhancers of the bacterial cellulose biofilm surface properties: an inverse chromatography approach. Ind. Crops Prod..

[7] Ma C. (2021). Wearable, ultrathin, and transparent bacterial celluloses/MXene film with Janus structure and excellent mechanical property for electromagnetic interference shielding. J. Chem. Eng..

[8] Wu Z. (2021). Oppositely charged aligned bacterial cellulose biofilm with nanofluidic channels for osmotic energy harvesting. Nano Energy.

[9] Siripongpreda T., Somchob B., Rodthongkum N., Hoven V.P. (2021). Bacterial cellulose-based re-swellable hydrogel: facile preparation and its potential application as colorimetric sensor of sweat pH and glucose. Carbohydr. Polym..

[10] Thaveemas P., Chuenchom L., Kaowphong S., Techasakul S. (2021). Magnetic carbon nanofiber composite adsorbent through green in-situ conversion of bacterial cellulose for highly efficient removal of bisphenol A. Bioresour. Technol..

[11] Sanay B., Streit S., Lima A., Margarete M. (2020). Bioactive bacterial cellulose membrane with prolonged release of chlorhexidine for dental medical application. Int. J. Biol. Macromol..

[12] Almeida A.P.C. (2022). Crosslinked bacterial cellulose hydrogels for biomedical applications. Eur. Polym. J..

[13] Płoska J., Garbowska M., Pluta A., Stasiak-Różańska L. (2023). Bacterial cellulose – innovative biopolymer and possibilities of its applications in dairy industry. Int. Dairy J..

[14] Huang W.-M. (2022). Immobilization of *Chlorella sorokiniana* AK-1 in bacterial cellulose by co-culture and its application in wastewater treatment. J. Taiwan Inst. Chem. Eng..

[15] Parvulescu O.C., Mondal Md I.H. (2021). Antimicrobial Textiles from Natural Resources.

[16] El-Gendi H., Salama A., El-Fakharany E.M., Saleh A.K. (2023). Optimization of bacterial cellulose production from prickly pear peels and its ex-situ impregnation with fruit byproducts for antimicrobial and strawberry packaging applications. Carbohydr. Polym..

[17] Revin V., Liyaskina E., Nazarkina M., Bogatyreva A., Shchankin M. (Nov. 2018). Cost-effective production of bacterial cellulose using acidic food industry by-products. Braz. J. Microbiol..

[18] Jozala A.F. (2016). Bacterial nanocellulose production and application: a 10-year overview. Appl. Microbiol. Biotechnol..

[19] Gao G., Liao Z., Cao Y., Zhang Y., Zhang Y., Wu M. (2021). Highly efficient production of bacterial cellulose from corn stover total hydrolysate by *Enterobacter sp*. FY-07. Bioresour. Technol..

[20] Lotfy V.F., Basta A.H., Abdel-monem M.O., Abdel-hamed G.Z. (2021). Utilization of bacteria in rotten Guava for production of bacterial cellulose from isolated and protein waste. Carbohydr Polym Technol Appl.

[21] Skiba E.A. (2020). A technology for pilot production of bacterial cellulose from oat hulls. Chem. Eng. J..

[22] Efthymiou M.-N. (2022). Property evaluation of bacterial cellulose nanostructures produced from confectionery wastes. Biochem. Eng. J..

[23] Li Z., Azi F., Ge Z., Liu Y., Yin X., Dong M. (2021). Bio-conversion of kitchen waste into bacterial cellulose using a new multiple carbon utilizing *Komagataeibacter rhaeticus*: fermentation profiles and genome-wide analysis. Int. J. Biol. Macromol..

[24] Hikal W.M. (2021). Pineapple (*Ananas comosus* L . Merr.), waste streams, characterisation and valorisation : an overview. Open J. Ecol..

[25] Cooray S.T., Lee J.J.L., Chen W.N. (2017). Evaluation of brewers' spent grain as a novel media for yeast growth. Amb. Express.

[26] Ketnawa S., Chaiwut P., Rawdkuen S. (2012). Pineapple wastes: a potential source for bromelain extraction. Food Bioprod. Process..

[27] Wichitsathian B., Yimrattanabavorn J., Wonglertarak W. (2020). Enhancement of biogas production from pineapple waste by acid-alkaline pretreatment. IOP Conf. Ser. Earth Environ. Sci..

[28] Nigam J.N. (2000). Continuous ethanol production from pineapple cannery waste using immobilized yeast cells. J. Biotechnol..

[29] Ueno T., Ozawa Y., Ishikawa M., Nakanishi K. (2003). Lactic acid production using two food processing wastes, canned pineapple syrup and grape invertase, as substrate and enzyme. Biotechnol. Lett..

[30] Lun O.K., Wai T.B., Ling L.S. (2014). Pineapple cannery waste as a potential substrate for microbial biotranformation to produce vanillic acid and vanillin. Int. Food Res. J..

[31] Algar I., Fernandes S.C.M., Mondragon G., Castro C., Garcia-astrain C., Gabilondo N. (2015). Pineapple agroindustrial residues for the production of high value bacterial cellulose with different morphologies. J. Appl. Polym. Sci..

[32] Hui C., Redza M., Rahman A., Idayu I. (2020). Optimization of bacterial cellulose production from pineapple waste using different fermentation method. Chem. Eng. Res. Des..

[33] Permatasari S. (2021). Atmospheric cold plasma-assisted pineapple peel waste hydrolysate detoxification for the production of bacterial cellulose. Int. J. Biol. Macromol..

[34] Faostat (2021).

[35] Meena L., Sengar A.S., Neog R., Sunil C.K. (2022). Pineapple processing waste (PPW): bioactive compounds, their extraction, and utilisation: a review. J. Food Sci. Technol..

[36] Polanía A.M., Londoño L., Ramírez C., Bolivar G., Aguilar C.N. (2023). Biomass conversion and biorefinery. Valorization of pineapple waste as novel source of nutraceuticals and biofunctional compounds.

[37] Kodagoda K.H.G.K., Marapana R.A.U.J. (2017). Sri Lanka Association for the Advancement of Science.

[38] Roha S., Zainal S., Noriham A., Nadzirah K. (2013). Determination of sugar content in pineapple waste variety N36. Int. Food Res. J..

[39] Srikandace Y. (2022). Bacterial cellulose production by *Komagataeibacter xylinus* using rice-washed water and tofu processing wastewater with the addition of sodium glutamate. Fibers Polym..

[40] Schramm M., Hestrin S. (1954). Factors affecting production of cellulose at the air/liquid interface of a culture of *Acetobacter xylinum*. Journal of Genetic Microbiology.

[41] Somogyi M. (1952). Notes on Sugar Determination.

[42] Dórame-Miranda R.F., Gámez-Meza N., Medina-Juárez L.Á., Ezquerra-Brauer J.M., Ovando-Martínez M., Lizardi-Mendoza J. (2019). Bacterial cellulose production by *Gluconacetobacter entanii* using pecan nutshell as carbon source and its chemical functionalization. Carbohydr. Polym..

[43] Blanco Parte F.G. (Apr. 2020). Current progress on the production, modification, and applications of bacterial cellulose. Crit. Rev. Biotechnol..

[44] Nguyen Q.D., Nguyen T.V.L., Nguyen T.T.D., Nguyen N.N. (Feb. 2022). Effects of different hydrocolloids on the production of bacterial cellulose by *Acetobacter xylinum* using Hestrin–Schramm medium under anaerobic condition. Bioresour. Technol. Rep..

[45] Urbina L., Corcuera M.Á., Gabilondo N., Eceiza A., Retegi A. (2021). A review of bacterial cellulose: sustainable production from agricultural waste and applications in various fields. Cellulose.

[46] Kongruang S. (2008). Bacterial cellulose production by *Acetobacter xylinum* strains from agricultural waste products. Appl. Biochem. Biotechnol..

[47] Khan H., Saroha V., Raghuvanshi S., Bharti A.K., Dutt D. (May 2021). Valorization of fruit processing waste to produce high value-added bacterial nanocellulose by a novel strain *Komagataeibacter xylinus* IITR DKH20. Carbohydr. Polym..

[48] Algar I. (Jan. 2015). Pineapple agroindustrial residues for the production of high value bacterial cellulose with different morphologies. J. Appl. Polym. Sci..

[49] Nguyen V.T., Gidley M.J., Dykes G.A. (2008). Potential of a nisin-containing bacterial cellulose film to inhibit Listeria monocytogenes on processed meats. Food Microbiol..

[50] Chibrikov V., Pieczywek P.M., Zdunek A. (2023). Tailor-made biosystems - bacterial cellulose-based films with plant cell wall polysaccharides. Polym. Rev..

[51] Sanchez S., Demain A.L. (2008). Metabolic regulation and overproduction of primary metabolites. Microb. Biotechnol..

[52] Kouda T., Naritomi T.I., Yano H., Yoshinaga F. (1997).

[53] Pacheco G., Nogueira C.R., Meneguin A.B., Trovatti E., Barud H. da S. (2017). Development and characterization of bacterial cellulose produced by cashew tree residues as alternative carbon source. Ind. Crops Prod..

[54] Xie X., Zhou Z., Jiang M., Xu X., Wang Z., Hui D. (2015). Cellulosic fibers from rice straw and bamboo used as reinforcement of cement-based composites for remarkably improving mechanical properties. Compos. B Eng..

[55] Gayathri G., Srinikethan G. (2019). Bacterial Cellulose production by *K. saccharivorans* BC1 strain using crude distillery effluent as cheap and cost- effective nutrient medium. Int. J. Biol. Macromol..

[56] Yassine F. (2016). Two-step formation mechanism of *Acetobacter* cellulosic biofilm: synthesis of sparse and compact cellulose. Cellulose.

[57] Salari M., Sowti M., Rezaei R., Ghanbarzadeh B., Samadi H. (2019). Preparation and characterization of cellulose nanocrystals from bacterial cellulose produced in sugar beet molasses and cheese whey media. Int. J. Biol. Macromol..

[58] Mohd Amin M.C.I., Ahmad N., Halib N., Ahmad I. (2012). Synthesis and characterization of thermo- and pH-responsive bacterial cellulose/acrylic acid hydrogels for drug delivery. Carbohydr. Polym..

[59] Atykyan N., Revin V., Shutova V. (2020). Raman and FT-IR Spectroscopy investigation the cellulose structural differences from bacteria *Gluconacetobacter sucrofermentans* during the different regimes of cultivation on a molasses media. Amb. Express.

[60] French A.D. (2014). Idealized powder diffraction patterns for cellulose polymorphs. Cellulose.

[61] Nguyen H.T., Saha N., Ngwabebhoh F.A., Zandraa O., Saha T., Saha P. (Sep. 2021). Kombucha-derived bacterial cellulose from diverse wastes: a prudent leather alternative. Cellulose.

[62] Soykeabkaew N., Sian C., Gea S., Nishino T., Peijs T. (2009). All-cellulose nanocomposites by surface selective dissolution of bacterial cellulose. Cellulose.

[63] Zhao H. (Mar. 2018). Production of bacterial cellulose using polysaccharide fermentation wastewater as inexpensive nutrient sources. Biotechnol. Biotechnol. Equip..

[64] Oliveira R.L. (Sep. 2015). Synthesis and characterization of methylcellulose produced from bacterial cellulose under heterogeneous condition. J. Braz. Chem. Soc..

[65] Hirata T., Nishimoto T. (1991). DSC, DTA, and TG of cellulose untreated and treated with flame-retardants. Thermochimica.

